# Small Things that Make a Big Difference: Single-Cell Transcriptomic of Nanociliates Reveals Genes Potentially Involved in Mixotrophy

**DOI:** 10.1007/s00248-025-02575-4

**Published:** 2025-07-09

**Authors:** Filomena Romano, Uwe John, Michele Laval-Peuto, Paraskevi Pitta

**Affiliations:** 1 Institute of Oceanography, Hellenic Centre for Marine Research, PO Box 2214, 71003 Heraklion, Greece; 2 Department of Ecological Chemistry, Alfred Wegener Institute (AWI), Helmholtz Centre for Polar and Marine Research, Bremerhaven, Germany; 3 Helmholtz Institute for Functional Marine Biodiversity at the University of Oldenburg (HIFMB), Oldenburg, Germany; 4 Faculté Des Sciences Nice-Valrose, Université de NICE, Valrose, France; 5 144 Chemin Des Campons, 06480 Le Colle Sur Loup, France

**Keywords:** Single-cell transcriptomic, Mixotrophic ciliates, Phylogenetics, Homologous genes

## Abstract

Nanociliates play an important role in the microbial food web of oligotrophic marine systems as grazers of picoplankton on one side, and as prey for microplankton, on the other. However, knowledge on their taxonomy, phylogeny, and trophic strategies is very limited, as well as their potential role as mixotrophs. In the present study, we investigated the transcriptomes of five marine planktonic nanociliates isolated from the Eastern Mediterranean Sea. Our aim was the following: (i) to characterize the phylogenetic placement of these cells using concatenated phylotranscriptomic and (ii) to identify genes potentially involved in mixotrophy by focusing on both photosynthesis and digestion-related genes (phagosome, lysosome). Phylogenetic reconstruction revealed that two cells clustered with Tintinnida, while the other three clustered with Oligotrichida. Reciprocal best hits (RHBs) BlastP analysis indicated the presence of genes related to photosynthesis across all the transcriptomes, while the detection of genes associated with phagosome, lysosome, and generic metabolic pathways provided a more informative insight into the mechanism of mixotrophy. These findings suggest that photosynthesis-related genes alone may not be sufficient indicators of mixotrophic potential in nanociliates and highlight the importance of considering additional cellular pathways involved in phagotrophy. Moreover, these transcriptomes will help to establish a basis for the evaluation of differential gene expression in Oligotrichida, Choreotrichida, and Tintinnida, and a step stone for mixotrophic investigation.

**Supplementary Information:**

The online version contains supplementary material available at 10.1007/s00248-025-02575-4.

## Introduction

Ciliates (phylum Ciliophora) are unicellular eukaryotes occurring in different habitats, from freshwater to marine aquatic environments, in soil or associated to other animals and plants [1, 2]. Ciliates are remarkable among other microorganisms because of unique biological characteristics, such as nuclear dimorphism, chromosomal fragmentation, and conjugation as sexual reproduction strategy. They usually dominate the microzooplankton (20–200 μm in length) group and they are major consumers of phytoplankton in aquatic ecosystems; thus, they are an important channel of energy transfer through the microbial food web [2]. As components of the ciliate community, nano-sized ciliates (< 20 μm in length) have also been recognized as a significant link in the marine microbial food web, because they are grazers of bacteria, pico-eukaryotes, and small phytoplanktonic groups in ultra-oligotrophic environments, such as the Eastern Mediterranean Sea [3, 4]. Nano-ciliate community is dominated by the two sister subclasses [3, 5] Choreotrichida (or choreotrichs) and Oligotrichida (or oligotrichs). These subclasses include heterotrophic and mixotrophic forms (the latter belonging only to Oligotrichida) [6, 7]; thus, they play different roles in the microbial loop (e.g., algae and bacteria consumers on one side, and primary producers on the other) [2]. The presence of mixotrophic nanociliates was reported in the Eastern Mediterranean Sea, dominating the whole mixotrophic ciliate community [2, 3]. The taxonomy of nanociliates is based on cell morphology and ciliary patterns, which are studied in vivo and by complex staining techniques, such as protargol staining, which is useful to reveal the infraciliature of ciliates [8]. Over the last decades, the taxonomy of ciliates has been implemented by DNA sequences retrieved with molecular methods. Initially, this approach was based on the analysis of 18S ribosomal RNA genes (rRNA genes), that revealed a wide and unsuspected diversity when first applied to marine plankton [9–13]. In addition, some ciliate transcriptomic data are available in the Marine Microbial Eukaryote Transcriptome Sequencing Project (MMETSP; http://marinemicroeukaryotes.org). Despite the implementation of ciliate taxonomy and systematic during the last decades, many nanociliate taxa remain undiscovered. One of the advantages of the single-cell transcriptomic is the possibility to investigate on the physiology and the availability of genes involved in many different patterns, such as metabolism, cell cycle, and most importantly, genes potentially involved in mixotrophy. Recent studies have made significant progress in understanding the genomics and the transcriptomics of marine mixotrophic ciliates. A study that was conducted in *Strombidium* cf. *basimorfum* showed that prey rRNA and other transcript of prey nuclear origin were transcriptionally active inside the ciliate [14]. On the other side, Santoferrara et al. characterized the transcriptomes of two marine planktonic ciliates, trying to identify genes potentially involved in mixotrophy. However, they were not able to establish which genes are clearly involved in chloroplasts maintenance and functioning in the transcriptome of the mixotrophic ciliate *Strombidium rassoulzadegani* [15]. In mixotrophic ciliates, several lineages exhibit keptoplasty, whereby ingested chloroplasts from algal prey are retained temporarily for photosynthesis rather than digested [6, 16–18]. This suggests that in mixotrophic (kleptoplastidic) ciliates, the phagosome-lysosome system may differ from that of strictly heterotrophic species, as the degradation of retained plastids is avoided in mixotrophs. This implies the possible presence of different isoforms of digestive enzymes that do not interfere with chloroplast digestion in mixotrophic ciliates. In the few studies that investigated on differential gene expression between mixotrophic and heterotrophic ciliates, genes related to phagosome, lysosome, and generic metabolic pathways are not taken into account, thus starting analyzing these pathways at the single-cell level offers a window into the molecular adaptations underlying mixotrohy, particularly regarding selective digestion and organelle retention. In this study, we investigated the transcriptomes of five nanociliate specimens isolated from the ultra-oligotrophic Eastern Mediterranean Sea, using Illumina RNA-seq. Our aims were focused on two specific points:

1. Understanding the phylogenetic placement of the nanociliates isolated from the three most abundant groups found in the Eastern Mediterranean Sea (Oligotrichida, Choreotrichida, and Tintinnida);
2. Identifying genes involved in mixotrophy, and in particular, to explore the hypothesis that additional genes involved in phagosome, lysosome, and generic metabolic pathways are different between mixotrophic and heterotrophic ciliates.

## Materials and Methods

### Sample Collection

Sample collection was performed at the port of Gouves (Heraklion, Crete, Greece; LAT: 35° 20′ 07.0″ N, LONG: 25° 16′ 53.6″ E) in October 2019 from the very surface layer. From 2 L of water collected with a plastic beaker, 32 cells were manually isolated with a Pasteur glass pipette under a stereoscope using × 8 magnification and bright field. The ciliates were recognized based on their smooth helical trajectories and rapid jumps swimming behaviors [19]. After initial isolation, each ciliate was carefully washed 3 times in sterile, 0.2 µm-filtered seawater using a fine-tipped glass micropipette. During each transfer, cells were visually examined under the stereoscope to ensure that only the target cell remained in the droplet, and no obvious contaminants were visible. After the washing step, cells were immediately transferred in a 0.2 mL PCR tube containing the Lysis buffer provided by the extraction RNAqueous™-Micro Total RNA Isolation Kit (Thermo Fisher Scientific, Waltham, MA, USA) and stored at − 80 °C.

### RNA Extraction, cDNA Preparation, and Transcriptome Assembly

Eleven samples out of 32 (which were the samples with the highest confidence in taxonomy) were defrosted and transferred in 0.5 mL tubes (Eppendorf, Hambourg, Germany) containing acid washed glass beads (Sigma-Aldrich, St. Louis, MO, USA). RNA extraction was performed using NEBNext Single Cell/Low Input RNA Library Prep Kit for Illumina (New England Biolabs, Ipswich, MA, USA) according to manufacturer protocol and the complementary DNA (cDNA) libraries were generated using the SMART-Seq v4 Ultra Low Input RNA Kit for Sequencing (Takara Bio Europe SAS, Saint-Germain-en-Laye, France), according to manufacturer’s protocol. cDNA was quantified using a Nanodrop (Thermo Fisher Scientific, Waltham, MA, USA). Adapter and index ligation was done using the Nextera® XT DNA Library Preparation Kit (Illumina, San Diego, CA, USA). The raw sequences were demultiplexed using bcl2fastq software for Illumina, and contamination from adapters was checked using fastQC [20]. Reads were trimmed with TrimGalore [21]. The assembled transcriptomes were annotated using Trinotate v3.0.2 and the top blastx hit with *e*-value ≤ 10e^−9^ was selected in order to increase the accuracy of protein assignment. The resulting PFAM domains were assigned to Kyoto Encyclopedia of Genes and Genomes (KEGG) orthologies for functional annotation, while ribosomal transcripts were predicted with RNAmmer [22]. When 18S and 28S rRNA genes were not detected through RNAmmer, a BlastN search was performed against the assembled transcriptome data using 18S and 28S rRNA genes detected with RNAmmer as a query. Genes with the lowest *E*-value, with > 90% alignment length and > 97% identity were taken into account. All the BlastN searches were performed using usegalaxy.org. To confirm the taxonomic assignment, results from BlastN were further blasted in NCBI using default parameters. The raw read sequences have been deposited at the Sequence Read Archive (SRA) portal under the project number PRJNA718746 and submission ID SUB15380090. The completeness of the transcriptomes was analyzed using BUSCO v5 analysis following odb10_alveolata database [23].

### Phylogenetic Analysis

For phylogenetic analysis, already existing alignments of 18S and 28S rRNA gene sequences were retrieved from Santoferrara et al. (2014) [24] and re-aligned together with our dataset using MAFFT v.7 [25] with default parameters. Four sequences from Hypotrichia group were used as outgroup. Three final datasets were obtained as follows: (i) 18S rRNA, (ii) 28S rRNA, and (iii) concatenated 18S and 28S rRNA genes. Concatenated dataset was obtained using the Geneious Prime software and sequences were concatenated together when possible. Phylogenetic analyses were carried out using two different inferences. On one side, maximum likelihood (ML) inferences were processed with RAxML through the Geneious Prime software [26] with the node support value obtained out of 1000 bootstraps. On the other side, Bayesian Inferences (BI) were produced with MrBayes [27] with 1 million generations running and trees were sampled every 100 cycles. The first 1000 trees were discarded as burn-in and the remaining trees were used to estimate the Bayesian posterior probability. For both ML and BI analyses, the GTR mode with invgamma rate was identified by JModelTest [28]. Inference values above 50 (bootstrap support) and above 0.50 (posterior probability) were considered good. Phylogenetic trees were then customized using FigTree v1.3.1. [29] and iTOL [30].

### Search for Proteins Associated with Photosynthesis and Digestion

For comparative transcriptome analysis, the transcriptomes of five ciliates belonging to the class Spirotrichea were obtained from the literature of MMETSP project (Table 1). Candidate coding sequences (CDS) within transcripts and corresponding amino acid sequences (AAS) were predicted using the TransDecoder [31] tool with default parameters (minimum protein length = 100) on usegalaxy.org [32]. To investigate potential molecular traits of mixotrophy, we focused on genes associated with both photosynthesis and phagotrophy by examining KEGG pathways related to photosynthesis (ko00195), phagosome (ko04145), lysosome (ko04142), and general metabolic pathways (ko01100). We performed a two-step reciprocal BlastP analysis to identify putative homologous genes. In the first step, MMETSP transcriptomes were used as queries against our assembled transcriptomes. The top hits from this initial search were then used for a reverse BlastP, with our transcriptomes serving as the query. Genes that were identified as the best matches in both directions were considered reciprocal best hits (RBHs). Only BlastP results with an *E*-value < 10⁻^5^, percentage identity > 80% in both directions, and amino acid length > 50 were retained as strong candidates for putative homologous genes.

**Table 1:** Data information of five ciliates from the MMETSP project used in this work. Trophic strategies were assigned according to Schneider et al. (2020) [33]

MMETSP_ID	Order	Family	Species	Trophic strategy	Principal investigator
MMETSP0123	Tintinnida	Ptychocylididae	*Favella ehrenbergii*	Heterotrophic	Denis Lynn
MMETSP0208	Oligotrichida	Strombidiidae	*Strombidium inclinatum*	Heterotrophic	Denis Lynn
MMETSP0449	Oligotrichida	Strombidiidae	*Strombidium rassoulzadegani*	Mixotrophic	George McManus
MMETSP0126	Choreotrichida	Strombidinopsidae	*Strombidinopsis acuminatum*	Heterotrophic	Denis Lynn
MMETSP0463	Choreotrichida	Strombidinopsidae	*Strombidinopsis* sp.	Heterotrophic	George McManus

## Results

### Transcriptomes and FASTA Statistics

Successful sequences were obtained only from five cells out of eleven isolated. Since we lack of taxonomic features, we cannot be sure about their taxonomical names; for this reason, we decided to name the specimens as Cell1, Cell3, Cell5, Cell6, and Cell9, respectively. After removing prokaryotic contaminants, the number of transcripts ranged between 15,884 for Cell9 and 45,713 for Cell1 (Table 2).

**Table 2:** Transcriptome statistics

	Cell1	Cell3	Cell5	Cell6	Cell9
Overall GC content	41.41%	40.93%	39.98%	48.46%	48.49%
Number of contigs	45,713	36,517	45,616	23,509	15,884
Mean contig length	1041	1208	1109	1289	1336
Contig max-length	9257	12,668	8917	10,421	8290
Contig N50	1313	1534	1415	1720	1705
AA sequences	43,578	35,621	49,477	24,138	17,305

TransDecoder detected 43,578 amino acid sequences for Cell1, 35,621 for Cell3, 49,477 for Cell5, 24,138 for Cell6, and 17,305 for Cell9.

**Fig. 1:**
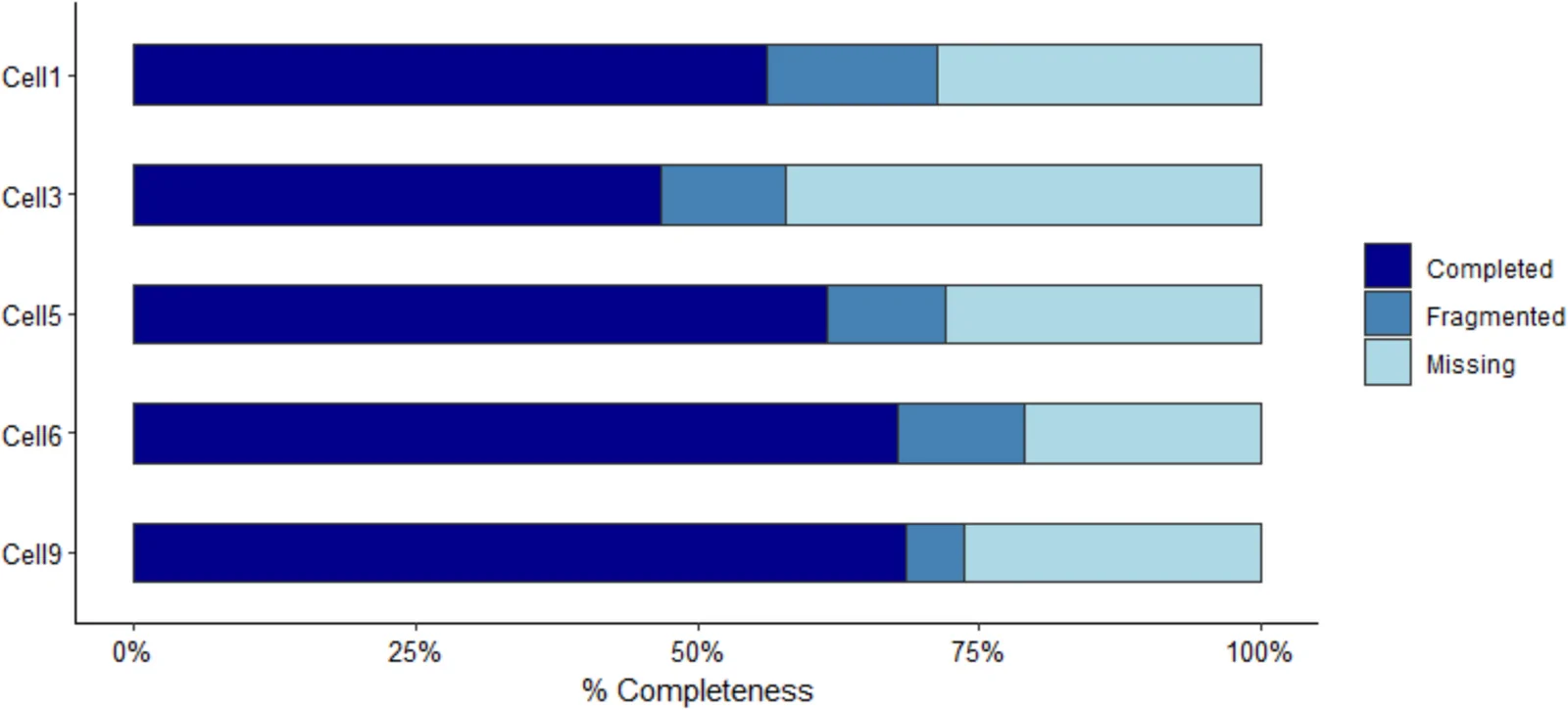
Results from BUSCO analysis on the five transcriptomes

The quality of the single-cell transcriptomes was shown by BUSCO analysis results. All the cells isolated yielded a transcriptome containing more than 50% of conserved genes in BUSCO coverage estimates against Alveolata database, with the exception of Cell3, which had the most complete coverage estimate of 46.80%, and a missing coverage of 42.1% of the transcriptome (Fig. 1).

### Functional Annotation

**Fig. 2:**
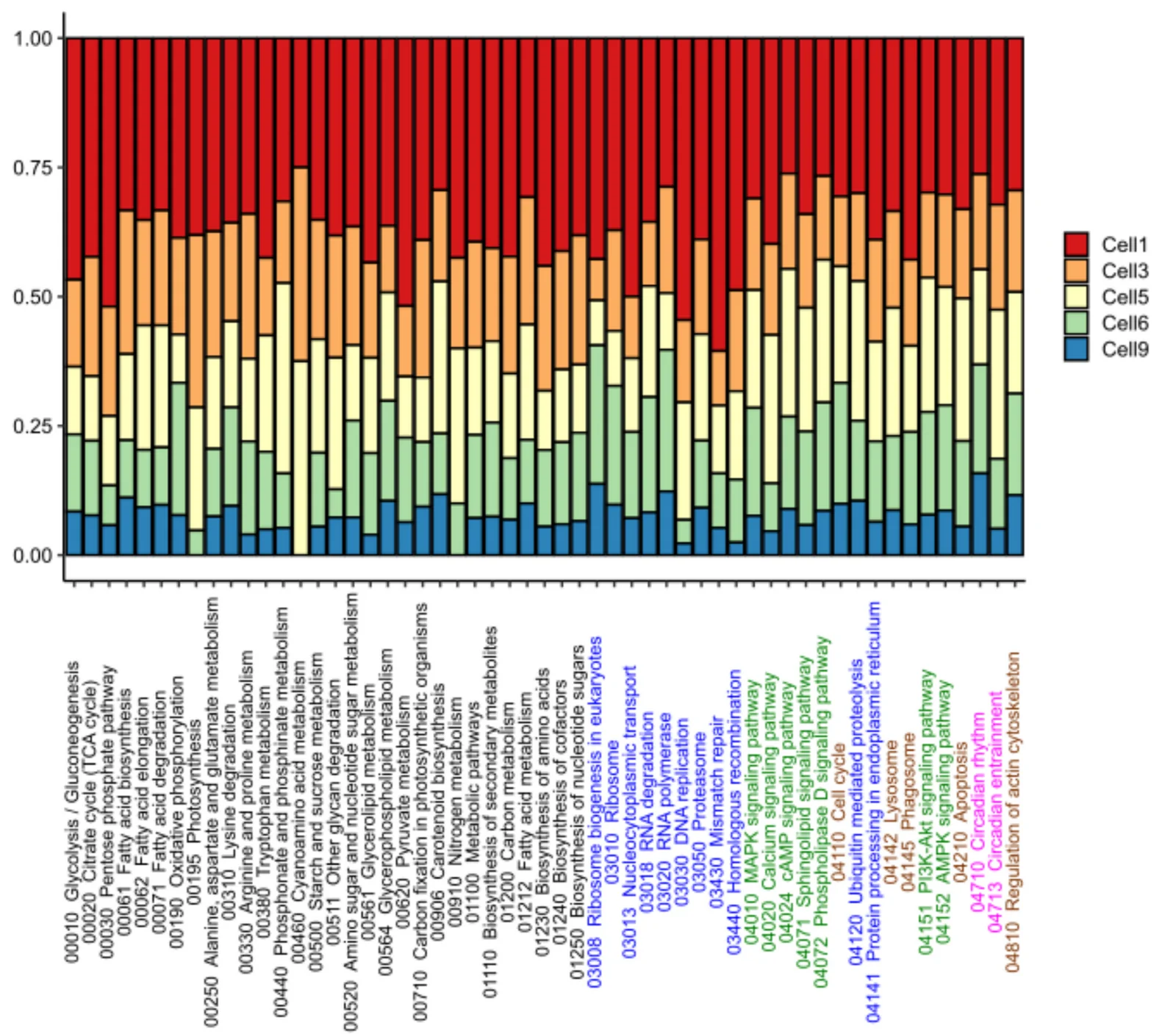
KEGG pathway distribution between nanociliates specimens collected in the present study. In black the annotation of genes related to Metabolism, in blue the annotations related to Genetic Information Processing, in green the annotation of genes related to Environmental Information Processing, in brown the annotations of genes related to Cellular Processes and in purple the annotation of genes related to Organismal Systems

The analysis of pathway annotations across the ciliates isolated in the present study revealed a diverse distribution of biological processes, with 5 major level 2 categories, 17 level 3 pathways, and 55 unique pathways (52 of which were common in all specimens). Among the level 2 pathway, “metabolism” was the most represented, accounting for an average of 63.77% of the total annotations, followed by “Genetic Information Processing” (16.49%), “Environmental Information Processing” (11.75%), “Cellular Processes” (6.93%), and “Organismal Systems” (1.04%, Table S1). The top level 3 pathways included “Global and overview maps” (44.10%), “Signal transduction” (12.27%), and “Translation” (8.68%), highlighting the dataset’s focus on signaling processes (Table S1). A broad overview of the functional annotations reveals that core cellular processes, such as general metabolism (01100 metabolic pathways), biosynthesis of secondary metabolites (01110), ribosome function (03010), and carbon metabolism (01200), are strongly represented and relatively consistent across all cells (both oligotrichs and choreotrichs). These pathways likely reflect fundamental, conserved functions necessary for basic cellular maintenance, growth, and protein synthesis. However, more specialized or condition-dependent pathways exhibit substantial variability across cells. In particular, pathways associated with photosynthesis and carbon fixation show a restricted distribution. For example, the “Photosynthesis” pathway (00195) and “Cyanoamino acid metabolism” (00460) were exclusively or predominantly detected in the oligotrichs Cell1, Cell3, and Cell5. In contrast, these pathways were absent or nearly undetectable in the choreotrichs Cell6 and Cell9. On the other hand, pathways related to cellular signaling (e.g., 04151 PI3K-Akt signaling pathway, 04120 Ubiquitin-mediated proteolysis, 04010 MAPK signaling), protein degradation (03050 Proteasome), and RNA processing (03020 RNA polymerase, 03018 RNA degradation) were more uniformly represented across all five cells. These pathways may represent generalized stress responses or regulatory mechanisms active in both groups regardless of trophic strategy (Fig. 2, Table S2).

### Phylogenetic Analysis

**Fig. 3:**
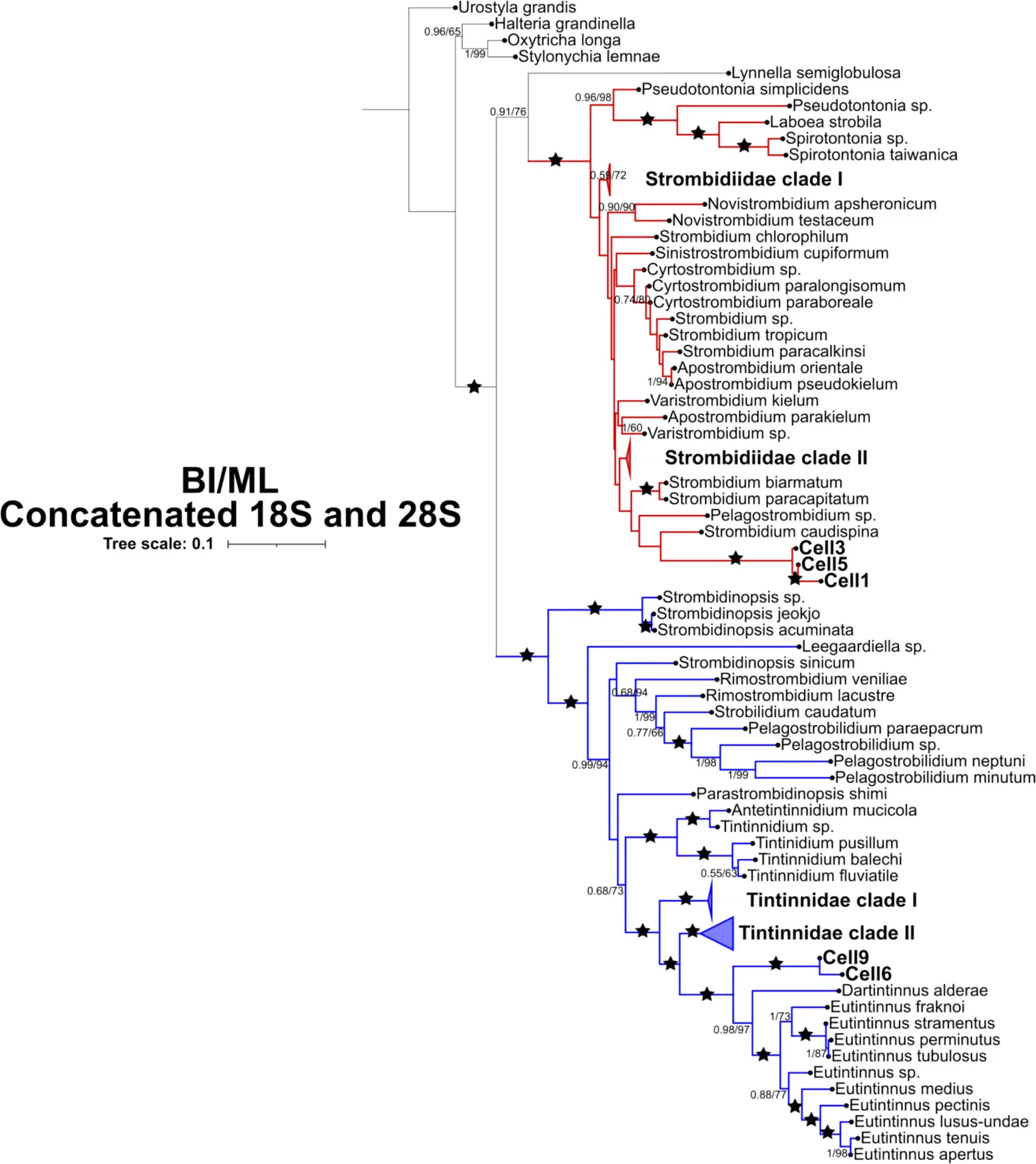
Phylogenetic tree of the ciliates using RAxML and MyBayes methods based on concatenated alignment of 18S and 28S rRNA genes from 207 representative species. Nodes with no values have less than 50% bootstrap support and less than 0.50 Bayesian Inference. The branch of oligotrichs is colored in red and the branch of choreotrichs is colored in blue. Nodes with 100% of bootstraps and posterior probability are spotted with black stars. The ciliates in the present study are in bold

Both the concatenated tree and 18S rDNA tree were topologically similar and most of their nodes were well supported by both ML and BI (Figs. 3, S1 and S2). ML and BI analyses showed similar supporting values for the concatenated tree and 18S. In all trees, the major three groups were recognizable: (1) Oligotrichida, represented by sequences Strombidiidae and Tontonnidae; (2) Choreotrichida, represented by Strobilidiidae; and (3) Tintinnida, represented by several tintinnids genera.

The trees built with both concatenated alignment and 18S rDNA, placed Cell6 and Cell9 in the Tintinnida clade with 100% and 1 bootstrap and posterior probability values, respectively (Figs. 3 and S1). In both trees, these nanociliates represented a sister clade of the *Eutintinnus* genus. On the other hand, Cell1, Cell3, and Cell5 clustered in the Oligotrichida clade, which was placed as sister clade of *Strombidium caudispina* in both concatenated and 18S rRNA gene alignments with 100% and 1 bootstrap and posterior probability values, respectively (Figs. 3 and S1).

### Homologous Genes Shared Within Already Known Ciliates from the Literature

We compared the amino acid sequences of the five ciliates analyzed in this study with datasets from the MMETSP project, which include the tintinnid *Favella ehrenbergii*, the choreotrichs *Strombidinopsis acuminatum* and *Strombidinopsis* sp. and the oligotrichs *Strombidium inclinatum* (heterotrophic), and *Strombidium rassoulzadegani* (mixotrophic). Using a reciprocal BlastP best-hit approach, we identified a total of 24,352 homologous genes. Specifically, Cell1 shared 7540 homologous genes in total with the other ciliates, while Cell3, Cell5, Cell6, and Cell9 had 4970, 5814, 3826, and 2202 homologous genes, respectively (Table 3).

**Table 3:** Number of homologous genes between the nanociliates specimens isolated in this study and the ciliates (class Spirotrichea) from MMETSP project. Maximum numbers are in bold, while minimum numbers are underlined

MMETSP	Cell1		Cell3	Cell5	Cell6	Cell9
*Favella ehrenbergii*		1026	607	763	650	411
*Strombidinopsis acuminatum*		**2178**	**1442**	**1553**	**1376**	**725**
*Strombidinopsis* sp*.*		1591	937	1063	862	525
*Strombidium inclinatum*		1416	1048	1312	506	306
*Strombidium rassoulzadegani*		1329	936	1123	432	235

All specimens exhibited the highest number of homologous genes with the choreotrich *Strombidinopsis acuminatum*. On the contrary, Cell1, Cell3, and Cell5 had the fewest homologous genes with the tintinnid *Favella ehrenbergii*, whereas Cell6 and Cell9 showed the lowest number of homologous genes with the oligotrich *Strombidium rassoulzadegani*.

Most amino acid sequences were shared among all specimens, while only a small percentage were orphan genes (sequences that did not have homologous in any of the reference datasets). The average percentage of orphan genes among the cells analyzed in this study was 10.13%.

**Fig. 4:**
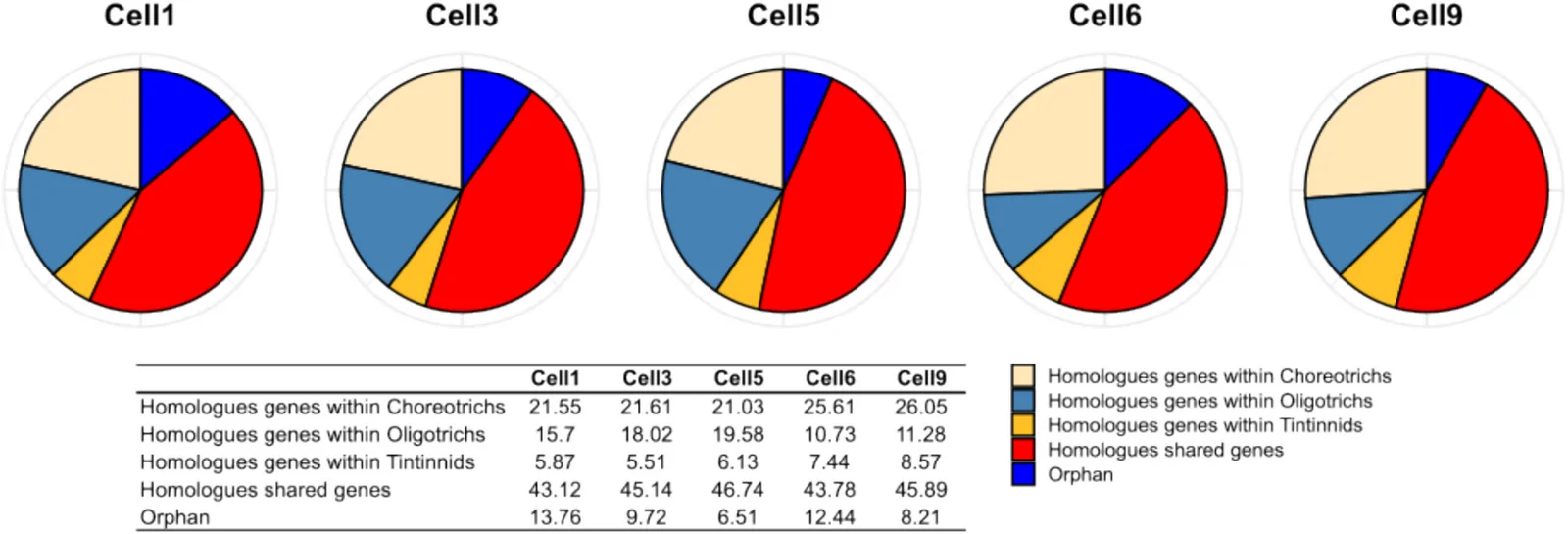
Best reciprocal BlastP hit distribution between the six nanociliates specimens of this study and the oligotrichs, choreotrichs and tintinnids from the MMETSP project. Orphan genes are those genes that did not match as homologs in any of the reference transcriptome. The tintinnids from MMETSP project is *Favella ehrenbergii*, the choreotrichs are *Strombidinopsis* sp. and *Strombidinopsis acuminatum*, while the oligotrichs are *Strombidium inclinatum* and *Strombidium rassoulzadegani*

On the other side, the average percentage of shared genes across all species was 44.93%. More specifically, the average percentage of homologous genes within oligotrichs, choreotrichs, and tintinnids was 15.06%, 23.17%, and 6.70%, respectively (Fig. 4). Among the ciliate specimens analyzed, Cell5 had the lowest percentage of orphan genes (6.51%), while Cell1 had the highest (13.76%). Additionally, all specimens exhibited a very low percentage of shared genes with the tintinnid *Favella ehrenbergii*.

### Are Specific Processes Such as Phagosome, Lysosome, and Some More General Metabolic Pathways Associated Enzymes Keys for Mixotrophy?

We examined the presence of homologous genes associated with the phagosome, lysosome, and core metabolism pathways between our single-cell transcriptomes and MMETSP transcriptomes from oligotrich, choreotrich, and tintinnid ciliates. Homologous genes involved in the phagosome pathway, such as *ATPeV1A*, *RAB5A*, and *SEC61A*, were broadly distributed across all groups, reflecting the conserved nature of phagocytosis in ciliates. However, *DYNC2H*, a gene involved in dynein motor function and intracellular transport, was exclusively present in oligotrich cells and absent from choreotrichs and tintinnids, suggesting possible lineage-specific differences in phagosomal trafficking mechanisms. Within the lysosome pathway, homologous genes (*AP1M*, *AP1S1*, *AP4E*, *AP4M*, and *AP4S*) were widely distributed among all cells. However, lysosomal enzymes encoded by *AGA* and *CTSZ* were only identified in oligotrich cells, more specifically homologous genes between our oligotrichs and the mixotrophic *Strombidium rassoulzadegani*, indicating possible functional specialization of lysosomal pathways within this group. (Table S3).

The comparative analysis of homologous genes involved in core metabolism among MMETSP ciliates and the ciliates from the present study reveals distinct patterns of gene presence across taxonomic groups. More specifically, genes shared between our oligotrich cells and the mixotrophic *Strombidium rassoulzadegani* included UDP-N-acetylglucosamine transferase *(ALG7)*, enoyl-CoA hydratase *(ECHS1)*, glutathione dehydrogenase* (frmA)*, glyoxylate/hydroxypyruvate reductase* (ghrB)*, mannose-1-phosphate guanylyltransferase *(GMPP)*, glucose-6-phosphate isomerase *(GPI)*, cAMP-specific phosphodiesterase 4 *(PDE4)*, phenylalanine-4-hydroxylase* (phha)*, ubiquinol-cytochrome c reductase *(QCR1)*, and UDP-sugar pyrophosphorylase (*USP*) (Table S4).

## Discussion

### Challenges of These Techniques

Initially, we successfully isolated 32 cells from the Eastern Aegean Sea through manual selection under a stereoscope. Then, we identified ciliates, among the isolated cells, based on morphology (presence of cilia) and their distinctive swimming behavior. This strategy, although effective, was technically demanding due to the small size, transparency, and fragility of the organisms. From the 32 isolated cells, we selected only 11 for transcriptomic sequencing (those for which we had the highest confidence in taxonomic identification based on live observations). This approach was necessary to reduce the risk of misclassification and to ensure the reliability of downstream analyses. In particular, it is possible that some of the 32 cells were dinoflagellates, and we preferred to sequence only those we were confident to be ciliates to avoid confusion with genes related to the photosynthesis.

Moreover, obtaining high-quality RNA from single ciliate cells is also challenging due to their limited cytoplasmic volume which increases the risk of RNA degradation during handling. Despite these technical limitations, the data generated in this study represent a valuable resource. Even though our approach remains limited in scale, we believe that these transcriptomic datasets can serve as a step-stone for future investigations into ciliate functional biology, gene diversity, and trophic strategies. As more refined techniques and broader datasets become available, our results may help guide comparative studies and improve understanding of the molecular basis of ecological roles among marine ciliates.

### Transcriptome Assembly

The two main findings from this study are (i) single-cell transcriptomics of nanociliates revealed that two out of five cells were unexpectedly clustered to tintinnids, while the other three in Oligotrichida, and (ii) genes related to mixotrophy may be involved not only in kleptoplasticity, but also in phagosome and lysosome. For all specimens, half of the totally assembled nucleotides were contained in sequences of 1313, 1534, 1415, 1720, and 1705 nt as indicated by the N50 values.

The BUSCO scores (50–60%) reflect challenges of de novo transcriptome assembly from environmental single-cell RNA-seq, as the limited material does not allow for the same sequence depth as for high RNA input cDNA libraries, furthermore in non-model organisms like pelagic ciliates that lack of reference genomes and functional annotation might be limited [23, 34]. For example, the sequenced genomes of ciliates are the ones of *Tetrahymena thermophila* [35], *Paramecium tetraurelia* [36], *Ichthyophthirius multifiliis* [37], and *Oxytricha trifallax* [38], so none of them is close to the nanociliates we isolated in our study. Furthermore, the genome of *Oxytricha trifallax*, that is the closest species to the Spirotrichea class, is fragmented into thousands of nano-chromosomes and it counts ca. 18,400 genes [38]. This has been a challenge for the few studies focused on pelagic ciliates, especially the ones from Oligotrichia family (which is the most neglected one compared to freshwater, or soil ciliate groups). Moreover, BUSCO analyses have been found to underestimate highly fragmented genomes [39]. Despite these challenges and even if the completeness of the transcriptomes might be considered moderate, the data contains a wide set of functionally informative genes and lineage-specific markers that support both the taxonomic identity and biological relevance of the data. For example, in all our transcriptomes, we still captured a substantial number of genes with functional annotations associated with metabolism, environmental sensing, and ribosome.

### Phylogenetic Clusters

The concatenated alignment between the two genes 18S, 28S rRNA, placed three out of five nanociliate specimens in the Oligotrichida group, while the other two clustered in Tintinnida group in all phylogenetic analysis. An interesting point for this study is that it was unexpected that two out of five cells were clustered in tintinnids, since the cells isolated were without lorica (data not shown). In nature, naked tintinnids, which were sorted among other aloricate ciliates, may have two different origins:

1. A proter, swimming in the field and disturbed during lorica building, may waste drops of lorica material and even lose its new growing lorica if its peduncle is badly attached. Such a naked proter of tintinnid, if surviving sufficiently, would/could produce new lorica material and build, finally and slowly, a coxlielliform lorica [40];
2. An opisthe detached from the original lorica is also possible in nature as well as in the lab. Depending on its age, it may contain or not nuclei with replication band and more or less granules of lorica material.

Since our nanociliates were without lorica, but they were phylogenetically close to tintinnids, it is possible that during the isolation we picked tintinnid proters or opisthes.

Another possible explanation for this unexpected result is the possibility for tintinnids to abandon their lorica in presence of stressful conditions, such as sampling with planktonic nets [2]. However, our sampling method did not involve any planktonic net or something that could induce so much stress to the tintinnids, so we discarded this hypothesis.

This information is important for the understanding of the complexity of ciliate taxonomy and phylogenetic placement. Indeed, pelagic ciliate taxonomy is exceptionally challenging due to the immense diversity and morphological plasticity of these microorganisms. They exhibit a wide range of shapes, sizes, and structural features that can change in response to environmental conditions. Traditional morphological identification methods are often insufficient, as many ciliates lack distinct and consistent features that can be easily observed under a microscope, for example coxlielliform loricae form (genetically similar but morphologically distinct loricae) [41]. Additionally, the high level of cryptic species (those that are genetically distinct but morphologically similar) further complicates taxonomic efforts [42–44]. Moreover, the dynamic nature of marine environments adds another layer of complexity, as ciliate populations can rapidly shift in response to changes in water temperature, salinity, and nutrient availability, making it difficult to obtain consistent and representative samples. For this reason, our results can be considered a step-stone for pelagic ciliate identification, due to the combination of different methods allowing the better understanding of ciliate taxonomy.

Another important aspect of the tintinnids without lorica, isolated for this study, is the confirmation of the presence of very small tintinnids in ultra-oligotrophic waters, such as the Eastern Mediterranean Sea. Very small tintinnids have been already described in the past, and some species have been already identified, such as *Codonella nationalis* [45], with a lorica size ranging from 15 to 23 μm in length.

Other small tintinnids were also found in the Arctic Ocean [46], suggesting that the possibility to find these kinds of ciliates is not low.

### Search for Protein Related to Photosynthesis

A kleptoplastidic organism engulfs photosynthetic prey and digests all but their chloroplasts [18]. The prediction of homologous genes between different species is a basic step in transcriptome studies, and it usually leads the identification of auxiliary genes among related organisms and the possibility to understand transcripts potentially related to mixotrophy. Transcripts for petC were not surprisingly found in the oligotrichs Cell5, *Strombidium rassoulzadegani* and *Strombidium inclinatum*. The petC gene encodes the Rieske iron-sulfur protein, a component of the cytochrome b6-f complex [47]. This complex plays a role in photosynthetic electron transport, mediating electron transfer between photosystem II and photosystem I, and is essential for photoautotrophic growth [47]. The fact that we detected the presence of this gene in the heterotrophic *Strombidium inclinatum* confirms the difficulty of using genes related to photosynthesis to understand genes related to mixotrophy in ciliates. Indeed, other studies that focused on genes related to photosynthesis were not able to discriminate which of the transcript could be strictly related to mixotrophy in ciliates [15]. This could be explained by the fact that these transcripts might be probably related to more general functions in a cell and not specifically to photosynthesis. Another explanation could be that the retained chloroplasts are simply stable and thus remain functional for some time, being autonomously active and can regulate themselves inside the host [14, 48]. For this reason, focusing on genes, related to photosynthesis and/or chloroplast to determine the transcripts potentially involved in mixotrophy, may be an insufficient approach.

### Proteins Related to Phagosome and Lysosome (Different “Digestive System?”)

As we explained above, the presence of transcripts potentially related to chloroplasts and photosynthesis might not be sufficient to understand the mechanism of mixotrophy in pelagic ciliates. For this reason, we think it is important to focus also on the mechanisms of digestion and metabolism. One difference between heterotrophic and mixotrophic ciliates is that chloroplasts survive to digestion, and they are transferred to the cytoplasm to be used as a source for carbon synthesis through photosynthesis [49]. Another difference is that mixotrophic ciliates have their chloroplasts irregularly arranged in the cytoplasm [6]; so they are somehow transported from the food vacuole to the cytoplasm through enzymes potentially involved in particle and vesicle transportation system. The most interesting result detected through best reciprocal hits BLASTp analysis, between the oligotrich Cell3 and the mixotrophic *Strombidium rassoulzadegani*, is the difference in the isoforms of cathepsin.

Cathepsins are a family of lysosome proteases that have been shown to play a significant role in marine protists [50]. For example, these enzymes facilitate the breakdown of ingested prey in heterotrophic flagellates [50]. Interestingly, among the examined ciliates, the mixotrophic *Strombidium rassoulzadegani* is the only species that possesses cathepsin Z (CTSZ), as a homologous gene with Cell3. This form is absent in all other species, including the heterotrophic *Favella ehrenbergii*, *Strombidinopsis acuminatum*, *Strombidinopsis* sp., and *Strombidium inclinatum*. This distinct enzymatic profile may provide a mechanistic explanation for the mixotrophic lifestyle of *Strombidium rassoulzadegani*. The presence of cathepsin Z, as opposed to cathepsin C (CTSC), which is more broadly detected, could suggest a more selective or regulated digestive strategy. Specifically, it is possible that cathepsin Z allows this species to avoid the complete degradation of ingested chloroplasts, thereby enabling the retention and use of functional chloroplasts. While this interpretation remains speculative, it highlights a potentially important link between digestion enzymes and trophic strategy. Moreover, it gives the information that it is possible that Cell3, isolated in the present study, may be mixotrophic as *Strombidium rassoulzadegani*.

### Search for Proteins Related to the General Classification of Metabolic Pathways

Beyond phagocytosis and digestion, several genes related to general metabolic pathways were found to be shared between oligotrich ciliates and the mixotrophic *Strombidium rassoulzadegani*, emphasizing the conservation of essential biosynthetic and energy production pathways. Among these genes, ALG7 is a gene related to N-glycosylation process available essentially in species belonging to Chlorophyta group, such as *Chlamydomonas reinharditii* and *Chlorella vulgaris* [2, 39, 41, 42, 51, 52]. Glycosylation is a process leading to the synthesis of oligosaccharides that are then attached to another molecule like a protein and they are involved in fundamental biological functions like cell adhesion, molecular trafficking and control of growth [53, 54]. It is possible that these glycans are related to molecular trafficking in mixotrophic species like *Strombidium rassoulzadegani* and the potential mixotrophs Cell1, Cell3, and Cell5, since chloroplasts are transported from the food vacuole to the cytosol after the process of keptoplasticity. Moreover, the gene ECHS1, involved in the catalysis of fatty acid metabolism, has been found only in the mixotrophic *Strombidium rassoulzadegani* and our oligotrichs. This enzyme is very important in microalgae metabolism because the fatty acid pathway provides microalgae with considerable energy for survival. While these genes are not uniquely linked to mixotrophy, their presence in potential mixotrophs and their role in energy production of microalgae underscore their potential relevance to supporting photosynthetic activity.

At last, we detected the gene GlyA (Cyanoaminoacid pathway) as homologous gene between our oligotrich and the mixotrophic *Strombidium rassoulzadegani*. The GlyA gene encodes the enzyme serine hydroxymethyltransferase (SHMT), which catalyzes the reversible conversion of serine to glycine, with tetrahydrofolate (THF) as the one-carbon carrier. This reaction is fundamental to one-carbon metabolism, contributing to the synthesis of nucleotides and amino acids. In photosynthetic organisms, SHMT is involved to the photorespiratory pathway, where glycine is converted to serine, a process that is closely linked to the Calvin cycle and essential for the efficient functioning of photosynthesis [55]. However, it is not known about the presence of this gene in photosynthetic plankton.

## Conclusion

Taken together, these findings provide evidence that oligotrich ciliates exhibit a high degree of metabolic differences, which may be an important factor in their ecological success. The presence of genes involved in phagocytosis, lysosomal digestion, and key metabolic pathways suggests that these ciliates are well-equipped to exploit a variety of nutritional resources, and this could be the first step for mixotrophy pathway identification. While most genes are not directly involved in photosynthesis, their functions in energy production, nutrient acquisition, and cellular regulation may play a crucial role in modes of nutrition in mixotrophic planktonic ciliates. Future research, including gene knockdown experiments and morphological identification, will be essential to determine the specific regulatory mechanisms that govern these pathways and to better understand how these organisms respond to environmental changes. Such investigations could provide further insights into the evolution of mixotrophy in marine protists and its implications for global biogeochemical cycles.

## Supplementary Information

Below is the link to the electronic supplementary material.

ESM 1 (XLSX 1.18 MB)

## Data Availability

The raw reads generated and analyzed during the current study are available in the Sequence Read Archive (SRA) portal under the project number PRJNA718746 and submission ID: SUB15380090.
